# Immersion Behavior of Carbon Steel, Phosphate Carbon Steel and Phosphate and Painted Carbon Steel in Saltwater

**DOI:** 10.3390/ma14010188

**Published:** 2021-01-02

**Authors:** Costica Bejinariu, Diana-Petronela Burduhos-Nergis, Nicanor Cimpoesu

**Affiliations:** Faculty of Materials Science and Engineering, Gheorghe Asachi Technical University, 700050 Iași, Romania; costica.bejinariu@tuiasi.ro

**Keywords:** seawater, corrosion resistance, paint, immersion behavior, phosphate layer, EIS, carbon steel, EDS, SEM

## Abstract

The carbon steel is used in many areas due to its good mechanical properties; however, its low corrosion resistance presents a very important problem, for example, when carbon steel carabiners are used in the petroleum industry or navy, the possibility of an accident is higher due to carabiner failure. This phenomenon could occur as a consequence of the corrosion process which negatively affects mechanical properties. This paper study the possibility to improve its corrosion resistance by depositing on its surface a phosphate layer and a paint layer, and also aims to analyze the immersion behavior in saltwater of carbon steel, phosphate carbon steel, and phosphate and painted carbon steel. According to this study, by coating the carbon steel with a phosphate or paint layer, a higher polarization resistance is obtained in saltwater. Moreover, by electrochemical impedance spectroscopy (EIS), it was observed that the corrosion rate decreases with the increase of the immersion time. Meanwhile scanning electron microscopy (SEM) and energy dispersive spectroscopy (EDS) revealed that the main compounds which formed on the sample’s surface were iron oxides or hydroxy-oxides, after immersion for a longer period. The overall results show that all types of deposited layers increase the corrosion resistance of C45 steel.

## 1. Introduction

Carbon steels, due to their good mechanical properties [1], as well as their possibility to be processed (welded [2], chipped [3], deformed [4]), are a suitable choice for use in the manufacture of machine parts [5], accessories of fall arrest systems (e.g., carabiners, hooks, and pythons) [6,7], vehicle bodies [8], shipbuilding [9], or in use in buildings, bridges, rails, pipes, [10] etc.

Compared to other metallic or non-metallic materials, carbon steels have a low corrosion resistance [11,12,13]. According to the literature, various methods have been attempted to improve the corrosion resistance of carbon steel; however, most of them are limited by the high manufacturing costs or complexity of the technologies. Nevertheless, one method which showed promising results was phosphate layer deposition on the surface [14].

According to previous studies [15,16,17,18], phosphating is one of the most widely used methods of protection against corrosion in metals [19]. The phosphating process consists of, with the help of a phosphating solution, a layer of zinc, iron, or manganese phosphate (depending on the major constituent of the phosphating solution) on the surface of the metal [20]. This layer is not only deposited on the surface, but also forms bonds with the material, with high adhesion to the substrate [21]. However, the quality and characteristics of the obtained phosphate layer highly depend on multiple parameters, such as the phosphating process parameters (stages, immersion time, and temperature), the characteristics of the substrate surface, the chemical composition of the phosphate solution (solution pH, type, and concentration of the metal ions), etc.

Accordingly, Schmidt D.P. et al. [22] studied the effect of the immersion time on the corrosion resistance of different types of commercial zinc-based coatings. Guenbour A. et al. [23] studied the corrosion resistance properties of steel on the surface, of which a layer of epoxy or chlorinated rubber was deposited, into which zinc phosphate was added. Asadi V. et al. [24] studied the immersion behavior for different periods of coated samples: phosphate with a zinc oxide solution. Moller H. et al. [25] studied the corrosion behavior of carbon steel immersed in natural and synthetic waters, highlighting chemical compounds that appeared on the surface using X-ray diffraction (XRD).

However, to our knowledge, there is no study which approaches the corrosion behavior evaluation of the carbon steel coated with Zn/Fe phosphate layer and phosphate-painted carbon steel immersed in a natural saltwater environment. Based on previous preliminary studies [17,26], the goal of this study is to analyze the influence of immersion time in saltwater of three types of samples: carbon steel, phosphate carbon steel, and phosphate and painted carbon steel, while a new phosphating solution suitable for carbon steel coating was introduced. Moreover, the corrosion mechanism and parameters for different immersion times were analyzed using EIS, SEM, and EDS.

## 2. Materials and Methods

### 2.1. Material

The steel used in this study is C45 carbon steel (Anterasteel, Targoviste, Romania), which has the chemical composition according to Table 1.

### 2.2. Sample Preparation

For the phosphate layer to cover evenly the entire surface of the steel, the phosphating was performed by an immersion comprising three main stages [27]. The first and second stages were performed to prepare the surface for the deposition of the phosphate layer. The surfaces of the samples were prepared by immersing them for 10 min in an alkaline degreasing solution (solution temperature: 85 °C ± 2 °C), after which they were immersed for 20 min in an acid pickling solution (at room temperature). A phosphate solution based on zinc and iron with the composition as shown in Table 2 was used to deposit the phosphate layer. Thus, the samples were immersed in the solution for 30 min at a temperature of 95 °C ± 2 °C. At the end of the phosphating process, the samples were dried for 4 h in an oven at 120 °C [17].

The paint used to cover the phosphate layer is a commercial paint, type KS 1000 (Car System GmbH, Uetersen, Germany). Its deposition was conducted by spraying [28].

In this study, to facilitate expression, the following notations were used:

C45—C45 steel sample;
PS—The C45 steel phosphate in zinc/iron-based solution sample;
PPS—The C45 steel phosphate in zinc/iron-based solution sample and painted;
BSW—Black Sea water, pH = 6.15.

### 2.3. Methods

The samples, in the form of discs with a diameter of 10 mm and a thickness of 2 mm, were immersed in closed bottles, in which 40 mL of corrosion medium (saltwater) was introduced. Weekly, the solutions were aerated by bubbling air. After 45 or 36 days, and 85 or 75 days, respectively, the samples were removed from the solution and analyzed using the method of electrochemical impedance spectroscopy (EIS) to study the structure of the surface layer and its evolution with the immersion time. The initial studies were performed one hour after immersion in solution (the time required for sample mounting operations in the electrochemical cell and thermostat) [26]. Only samples kept in saltwater for 85 days were used to study the surface morphology. These were removed from the solution, air-dried, and analyzed by scanning electron microscope with the energy of electron gun of 30 kV (SEM—VegaTescan LMH II, SE detector—Scanning Electron Microscope) (VegaTescan, Brno, Czech Republic), evaluated for surface micro-composition by the EDS method (Bruker detector) (Bruker, Berlin, Germany).

The visual analysis of the sample/solution systems indicated that the long contact between the alloy and the corrosion medium produces important changes, both in the surface structure of the samples and in the properties of the corrosion environment.

Some of the corrosion products can be soluble and pass into solution, changing its pH, and other parts, insoluble, remain on the surface or passed into solution if they are not adherent to the metal surface. In the case of the systems studied, the corrosion products were oxy-hydroxides (FeO(OH)) or hydroxides (Fe(OH)_2_, Fe (OH)_3_)) of iron. They are insoluble and poorly adherent to the surface, so that in the absence of a reciprocal solution/alloy displacement (liquid jet or alloy displacement through the liquid), part of it passing into the solution and part of it remaining on the surface. The remaining products on the surface are yellow-brown, porous, and slightly adherent to the surface.

**Figure 1:**
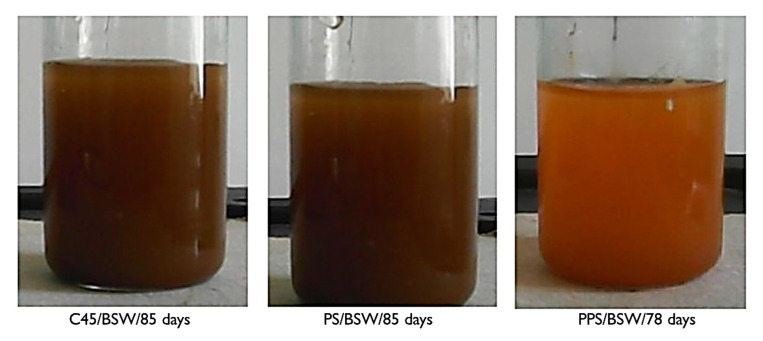
The corrosion environment appearance, after long immersion of alloys.

The products that passed in the corrosion environment were in the form of reddish-brown flakes, which in time are deposited at the bottom of the vessel, and the soluble products make the color of the seawater turn yellow. To observe, qualitatively, the changes of the corrosion environment, Figure 1 shows the physical aspect (the color) of the corrosion environment, after the samples were kept in solution for a long period. Prior taking the images, the solutions were stirred, in order to disperse the product from the bottom of the vessel.

In the case of seawater, the color intensity is proportional to the amount of insoluble reaction product passed into the solution.

Soluble corrosion products, but also products resulting from the degradation of coatings (phosphate layer, paint), have changed the pH of the corrosion environment, as can be seen from Table 3. The pH measurements were performed with an and pH-METER OP-2641/1 (Radelkis, Budapest, Hungary).

In all cases, a relatively small solution pH increased with the immersion time of the samples. This phenomenon occurs due to the reduction of H^+^ ions, which increases OH^−^ ion concentration from the solution [29]. Compared to the C45 sample, the PS and PPS samples produce a greater change in pH in seawater, probably because in this corrosion environment, the coating layers (phosphate layer and paint) are damaged.

The structure and composition of the surface layer were analyzed by electrochemical impedance spectroscopy (EIS) and scanning electron microscopy, as well as EDS microanalysis.

The EIS studies were performed with the PGZ 301 potentiometer (Radiometer Analytical SAS, Lyon, France), and the acquisition and processing of experimental data were performed with VoltaMaster 4 software (Version 7, Radiometer Analytical SAS, Lyon, France). The experimental data were processed with the ZSimpWin program (Version 3.5, E-chem Software, Ann Arbor, MI, USA), in which the spectra were interpreted by the fitting process developed by Boukamp, the least-squares method. To process, with this program, the data acquired with the VoltaMaster 4 program, the data were converted using the EIS file converter program. (Version 1.5, Radiometer Analytical S.A, Lyon, France) [30].

In this study, a three-electrode glass cell, C145/170 type (Radiometer Analytical SAS, Lyon, France), with a platinum auxiliary electrode (exposed surface area of 0.8 cm^2^), a reference electrode made of saturated calomel, and a 10 mm round sample of 3 mm thickness (exposed surface area of 0.503 cm^2^), fixed by Teflon washers on working electrode, was used. Furthermore, all the measurements were performed in the 104–25 × 10^−2^ Hz frequency range, at a sinusoidal potential amplitude of 10 mV. In the present study, the “ZSimWin” program was used for the analysis of impedance data. The program uses a wide variety of electrical circuits to numerically correlate the measured impedance data. The program can analyze very complicated dispersion data by decomposing the complex response into that of simple subcomponents. This approach, combined with the general procedure of nonlinear least squares correlation, allows the construction of an equivalent circuit whose simulated response has a high degree of correlation with the measured data. Depending on the frequency phase angle, one or more time constants may occur, and these can be used to determine the value of the parameters in the equivalent circuit. The presence of a compact passive film can be indicated from the Bode spectrum if the phase angle is close to 900 over the high-frequency range and if the spectrum has linear portions at intermediate frequencies.

The surface structure, both for freshly ground samples (C45 only) and for electrochemically corroded samples, was studied with a Vega Tescan LMH II electron microscope (30 kV, SE detector, high pressure).

The chemical compositions on various surface areas of the electrochemically untreated alloys and the surface of the corroded samples were evaluated using an EDX QUANTAX QX2 detector manufactured by Bruker/Roentec Co., Berlin, Germany EDS connected to the electron microscope.

To confirm the identification of the main compounds from the corrosion products, the rust crust, formed on the surface of the alloy, the oxides layers from the corroded samples was detached, dried in an oven, and analyzed by spectrophotometry. A BOMEM MB 104 FT-IR spectrophotometer (ABB Training Center GmbH & Co., Berlin, Germany) was used. The dry sample was dispersed in KCl (6% *w*/*w*), ground, and compressed into a disk at 10,000 Psi, and then scanned at a resolution of 4 cm^−1^ in the wavelength range 400–4000 cm^−1^.

## 3. Results and Discussion

### 3.1. The Effects of Prolonged Immersion of C45 in Black Seawater

**Figure 2:**
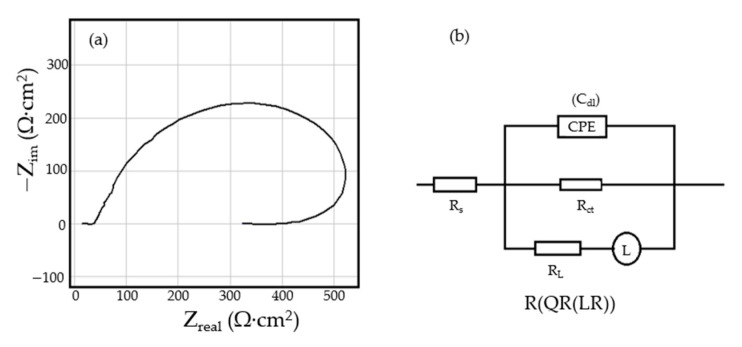
The Nyquist diagram (**a**) and equivalent circuit (**b**) for C45 sample, after one hour of immersion in BSW.

**Figure A1:**
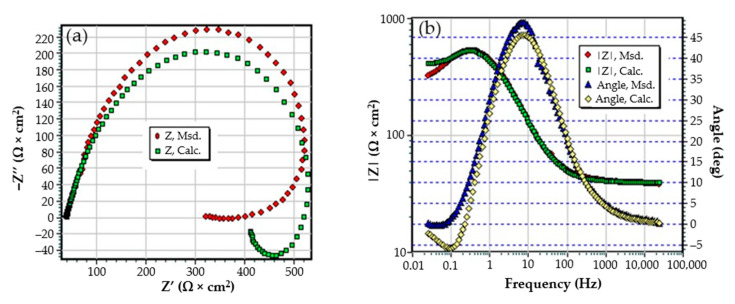
The Nyquist (**a**) and Bode (**b**) diagram for C45 sample after 1 h of immersion in BSW.

**Figure A2:**
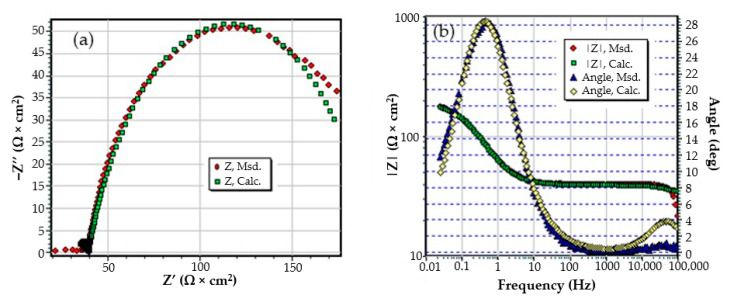
The Nyquist diagram (**a**) and Bode diagram (**b**) for C45 sample after 43 days of immersion in BSW.

**Figure A3:**
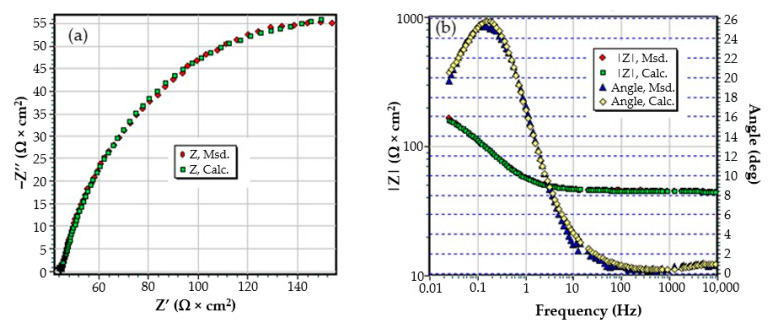
The Nyquist diagram (**a**) and Bode diagram (**b**) for C45 sample after 85 days of immersion in BSW.

**Figure A4:**
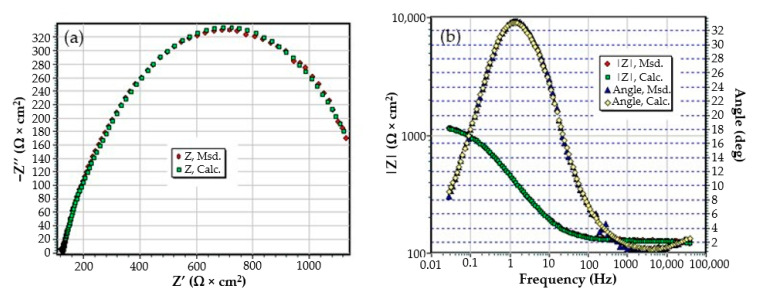
The Nyquist diagram (**a**) and Bode diagram (**b**) for PPS sample after 1 h of immersion in BSW.

**Figure A5:**
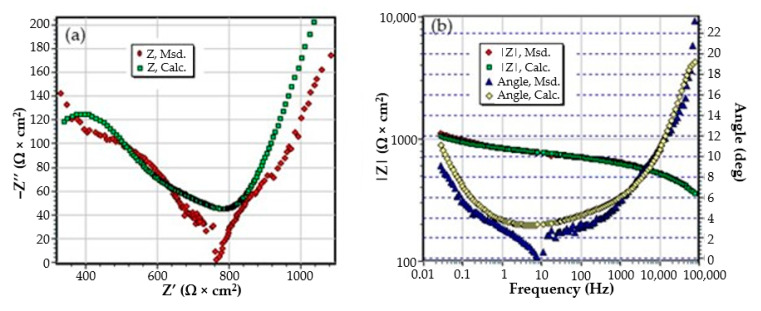
The Nyquist diagram (**a**) and Bode diagram (**b**) for PPS sample after 36 days of immersion in BSW.

**Figure A6:**
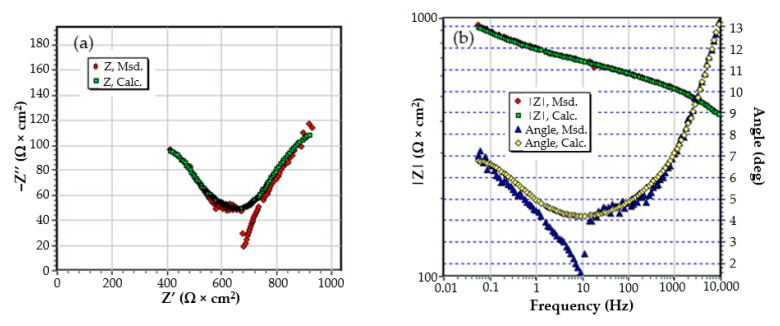
The Nyquist diagram (**a**) and Bode diagram (**b**) for PPS sample after 78 days of immersion in BSW.

The Nyquist diagram for the C45 sample, after 1 h of immersion in seawater, shows a depressed semicircle in the high-frequency range and an inductive loop in the low-frequency range (Figure 2a). The Nyquist and Bode diagrams for all the samples are presented in Figure A1, Figure A2, Figure A3, Figure A4, Figure A5 and Figure A6 from Appendix A. The low-frequency inductive loop is attributed to the adsorption processes of ions or neutral molecules (e.g., inhibitors) or even particles (may also be insoluble corrosion products) in the solution, but may also indicate the existence of intermediates reactions related to the adsorption of aggressive chlorine ions, which produce instability of the electrode surface [31,32].

The processing of the experimental data for this system was conducted with the equivalent electrical circuit from Figure 2b. Better adjustment (fitting) of the data was obtained by replacing the capacity of the double layer with a constant phase element (CPE), which expresses the ideal behavior of the capacity of the electric double layer (change of capacity with frequency). The equivalent circuit consists of a resistor (R_s_) in a series with a parallel combination of a constant phase element (CPE), a resistor (R_ct_), and a series resistor-inductance combination (R_L_, L) [26]. The value of the solution resistance (R_s_) is 38.67 Ω∙cm^2^, the value of the charge transfer resistance (R_ct_) is 588 Ω∙cm^2^, and the value of the resistance due to the adsorbed components (R_L_) is 1007 Ω∙cm^2^.

The low value of the R_ct_ indicates a high corrosion rate (compared to phosphate samples, R_ct_ exhibited higher values). For this circuit, the polarization resistance is calculated with the formula:

(1) $$R_{p}=\left(R_{L}\cdot R_{\mathrm{ct}}\right)∕\left(R_{L}+R_{\mathrm{ct}}\right)$$

and the R_p_ value is 377.23 Ω∙cm^2^. On the surface of the carbon steel sample, a continuous layer had not formed; instead, this was adsorbed on its surface ions from the saltwater or insoluble corrosion products.

**Figure 3:**
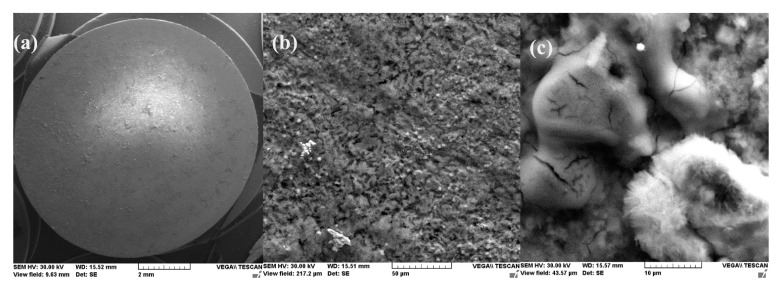
SEM microphotographs for C45 immersed 24 h in seawater (**a**) 100×, (**b**) 1000× and (**c**) 5000×.

This physical condition is confirmed by the microscopic and compositional analysis performed on a sample maintained for one day in seawater. Figure 3 shows the surface sample micrographs at various degrees of magnification. It was found that the surface of the sample was uniform and small crystalline formations were adsorbed on it, but these were reduced in number.

**Figure 4:**
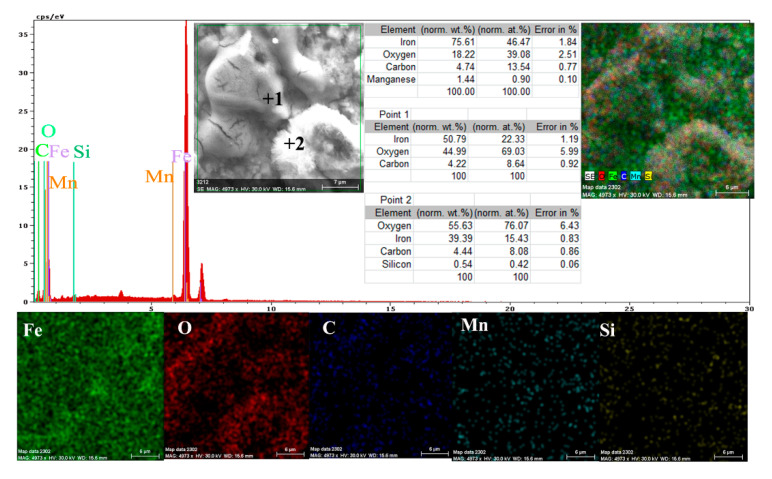
EDS spectrum, compositions and distribution of elements over a portion of the sample surface (Figure 3c).

A more conclusive figure (Figure 4) was provided by the EDX determinations on a small portion of the surface (850 µm^2^), on which the overall surface composition and point compositions on the main table and crystalline micro compounds were measured.

In the surface composition, oxygen and iron predominate formed a very thin and irregular film on the surface. Due to the very small thickness of this layer, the composition contained very small amounts of manganese and silicon, which were part of the main mass of the alloy. Surprising, however, was that a large amount of carbon (which, in the base alloy, is only 0.45%) and a large amount of oxygen (much more than the corresponding iron oxides) was observed. This anomaly could be explained when it is considered that HO^−^ ions are involved in the corrosion process (Fe + 2H_2_O→Fe^2+^ + 2OH^−^ + H_2_), in addition to carbon dioxide from the atmosphere. Chen et al. [28] explain the appearance of the inductive loop through a relaxation process, at the level of the surface layer, due to carbon monoxide and some HO^−^ anions in the solution.

**Figure 5:**
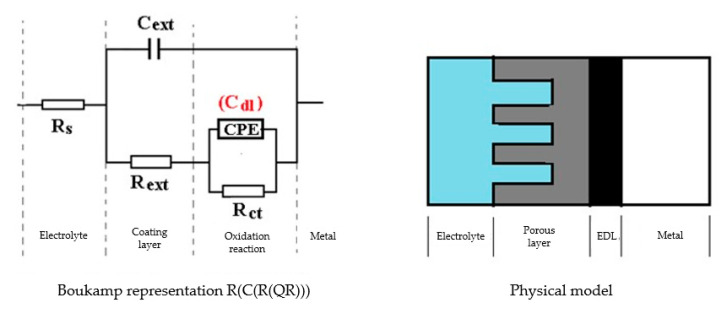
The equivalent circuit for C45 sample, after 43 days of immersion in BSW.

**Figure 6:**
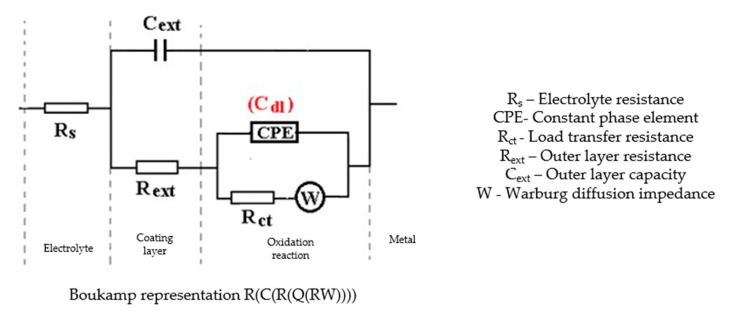
The equivalent circuit for C45 sample, after 85 days of immersion in BSW.

The Nyquist diagram, the Bode diagram, and the equivalent circuit used for the C45 sample immersed for 43 days in seawater is shown in Figure 5, as well as for the sample immersed for 85 days in Figure 6. The physical significance suggested by this circuit is the association of a film/electrolyte interface (R_ext_, C_ext_) with a kinetic process (R_ct_, C_dl_), R_ct_ being the charge transfer resistance through the electric double layer, and C_dl_ being the capacity of this layer. The R_ext_ resistor represents the resistance of the charge transfer through the open pores of the oxide layer, formed on the surface of the alloy, and the constant phase element C_ext_ represents the electrical capacity of this layer.

In the case of the sample kept for 85 days in seawater, the bi-layer structure is maintained, as in the case of the sample kept for 43 days in solution; however, for a better adjustment of the experimental data, it was necessary to introduce a Warburg impedance in series with charge transfer resistance. The diffusion impedance can be represented, analogously to the CPE impedance, by the relation [32]:

(2) $$Z_{W}=\frac{1}{w{\left(j\omega \right)}^{1∕2}}$$

The equation applies to linear diffusion, the unit of measure for W being: <Q> = Ω^−1^ sn^½^/cm^2^ ≡ S∙s^½^/cm^2^.

By this, the transfer of charges, to and from the surface of the alloy, is controlled both kinetically (through the electric double layer (controlled by R_ct_ and C_dl_), and by diffusion through the layer of products deposited on the surface. The values of the circuit elements parameters, for the two time periods considered, are presented in Table 4.

The analysis of the data allows us to advance a series of observations, as follows:

- The resistance of the solution (the electrolyte) increases slightly with the immersion time of the samples in seawater. This is because, in addition to the actual resistance of the liquid, there is also the physical resistance of the layer of corrosion products deposited on the surface. The strength of the product layer is very low because it is porous, non-adherent, and soaked in liquid.
- The resistance of the outer porous layer increases over time, due to the increase of its thickness, and accordingly decreases its electrical capacity.
- The constant Q, from the expression of the constant phase element that replaces the capacity of the double-electric layer, increases greatly with the immersion time (3.418·× 10^−4^—after 1 h, 6.92 ×·10^−3^—after 43 days and 1.25 ×·10^−2^—after 85 days). As a result, the impedance of this element increases greatly (according to the calculation relationship of ZCPE (CPE impedance)). The C_dl_ deviation from the idealist (expressed by the value of the exponent n) is large and is due to the increase in roughness due to corrosion.
- The layer of products, deposited on the surface, acts as a screen and causes the charge transfer resistance to decrease over time.
- The constant W, from the expression of the diffusion impedance, has a relatively low value and is manifested only in the case of the sample maintained for 85 days in seawater. As a result, the diffusion impedance is high and opposes the charge transfer along with R_ct_, thus reducing the corrosion rate over time.

**Figure 7:**
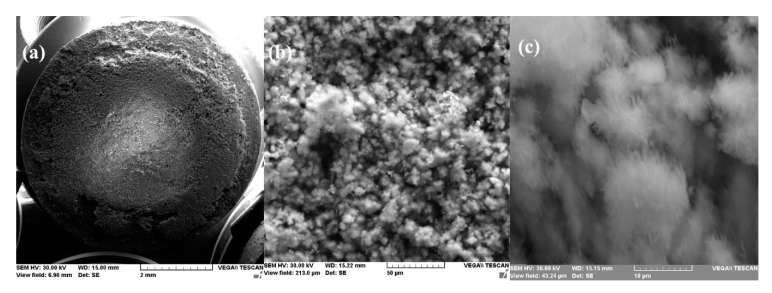
SEM microphotographs for C45 immersed 85 days in seawater (**a**) 100×, (**b**) 1000×, and (**c**) 5000×.

The surface appearance of the sample immersed 85 days in seawater, after removal from solution and drying, is shown in Figure 7 at various magnifying powers.

A thick layer of the reaction product was deposited on the surface of the sample forming a reddish-brown crust, very easily removable from the surface. Increased 1000×, the surface of the sample appears as an agglomeration of small crystalline and non-crystalline formations. The crystalline formations are better visible at a 5000× magnification and appear as an agglomeration of monoclinic crystals, characteristic for iron oxy-hydroxide.

**Figure 8:**
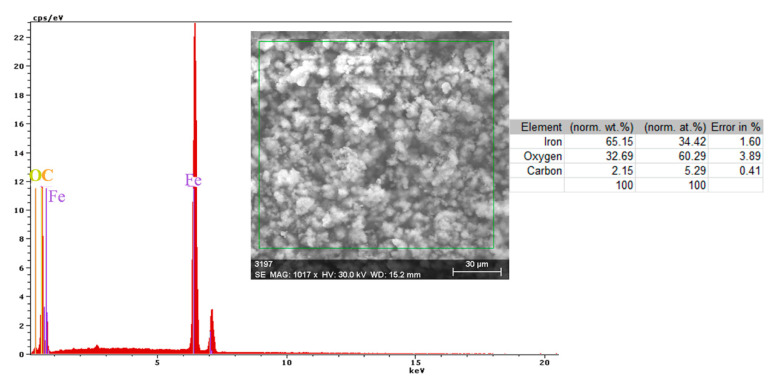
Energy spectrum and crust composition on the C45 surface immersed for 85 days in seawater.

**Figure 9:**
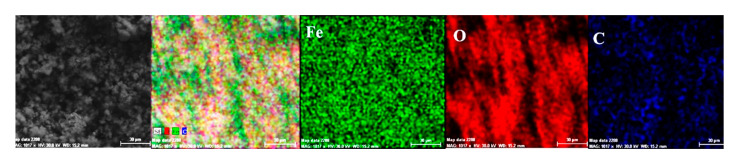
Elemental distribution on the surface of the crust of reaction products in the case of the C45/BSW system, after 85 days of immersion.

Both the energy spectrum (Figure 8) and the surface composition indicate only the presence of iron and oxygen, along with small amounts of carbon. In this composition, the molar ratio iron/Oxygen is very close to the value of 0.5 that is specific for ferric oxy-hydroxide (FeO(OH)), namely: n(Fe):n(O) = 0.57. Correlated with the type of crystals (monoclinic crystals), it can be considered that the entire surface is covered with a thick layer of FeO(OH). The presence of carbon could be attributed to the carbon dioxide absorbed in the crust. The distribution of the elements in the crust is shown in Figure 9. Areas with thick and large oxides cover parts of the surface (Figure 9), decreasing extensively the substrate iron signal. This layer plays a protective role for a while and after that, based on the continuous process of corrosion, the degradation of the material occurs. The presence of bicarbonate compounds in seawater leads to the formation of a carbon-based compound on the surface, starting from CO_3_ reactivity. The carbon presence was observed only on a few common parts of the oxygen distribution (Figure 9), which means that only a part of the surface compounds is complex.

An interesting approach is connected to the relationship between the mineral compounds of the corrosion environment (Black Sea water) and corrosion mechanisms of Fe-C alloys elements, with a focus on the effects of chloride and sulfate ions.

In our case, we strictly analyzed the experimental results, but further considerations can be realized. At the same time, we must consider various seawater parameters that affect the corrosion of iron-based elements; other chemical elements were not identified on the surface corroded crust. These parameters include pH value variation, oxygen dissolved in solution, and the number of cations and anions [33]. The influences of all these parameters will be analyzed in further works.

**Figure 10:**
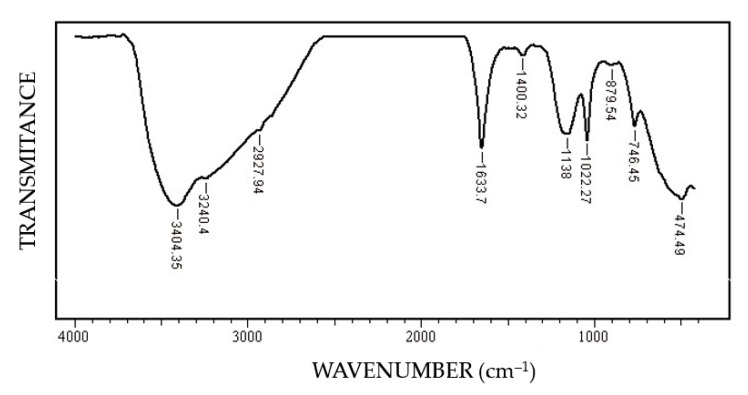
FT-IR spectrum of the corrosion product on the surface of the C45 sample maintained for 85 days in BSW.

In this FT-IR (Fourier-Transform Infrared Spectroscopy) spectrum (Figure 10) of the corrosion products, the characteristic bands FeOOH [34]: ν (OH) at 3402–3406 cm^−1^, δ (HOH) at 1633 cm^−1^, δ (OH) at 1400 cm^−1^, ν (FeO) at 474.49 cm^−1^ specific to steels were highlighted. The presence of the band 1633.7 cm^−1^ indicates the polymolecular structure of iron oxyhydroxide, formed by the interactions between FeOOH and H_2_O molecules.

### 3.2. Effects of Immersing C45 Phosphate (PS) in Black Sea Water

The C45 samples were phosphated in a solution based on Zn_3_(PO_4_)_2_ + Fe and were kept in Black Sea water for 43 and 85 days, respectively. After these periods, the samples were removed from the solution and introduced directly into the measuring cell, for the purpose of recording electrochemical impedance spectroscopy data. Another sample, kept in seawater for 85 days, was removed from the solution, dried in air, and analyzed. The measurements were performed after one hour of immersion (after mounting and thermostatic the sample).

The values of the equivalent circuit parameters for the three immersion periods (1 h, 43 days and 85 days) are presented in Table 5.

From the data analysis, the following can be highlighted:

- Although a layer (phosphate layer) is already deposited on the surface of the alloy, and after various periods another layer (corrosion products) is deposited on it, only a circuit with two constants was needed to correlate with the experimental data, considering a bi-layer structure of the surface (a passive layer-electric double layer and an outer layer). The outer layer consists of the phosphate layer and the corrosion products layer, very well intertwined, by occupying the pores of the phosphate layer with corrosion products;
- In this case, the resistance of the electrolyte (Rs) does not change with immersion time, the values obtained oscillating around the average value (40.58 Ω∙cm^2^), within the limit of experimental errors. This is because the outer layer is very porous (slightly permeable to solution) and has low resistance;
- After the first immersion period (43 days), the strength of the outer layer decreases instead of increasing. This could be due to the fact that the seawater partially damages the phosphate layer, widening its pores, and the corrosion products layer is not yet quite consistent. However, after 85 days of immersion, the resistance of the outer layer appreciably increases, due to the clogging of the pores of the phosphating layer and the thickening of the product layer;
- As in the case of the base alloy (C45) immersed in seawater, the resistance of the double-electric layer (charge transfer resistance) decreases with the immersion time in the corrosion environment, due to the screen effect of the outer layer;
- In the initial moments and in the period of up to 43 days of immersion, the corrosion process is controlled only kinetically, while at long immersion periods, the corrosion is controlled both kinetically and by diffusion;
- The capacity of the double-electric layer (C_dl_) is replaced here by a constant phase element, which takes into account the variation of the electrical capacity of the double layer with frequency. The values of the frequency exponent fact that in these cases the electric double layer behaves like a non-ideal capacitor.

**Figure 11:**
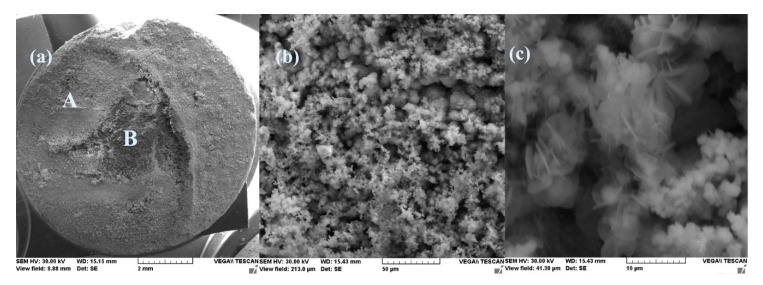
SEM microphotographs of the PS sample maintained for 85 days in seawater (**a**) 100×, (**b**) 1000×, and (**c**) 5000×.

The appearance of the sample surface and some of its details for various magnifications are illustrated in Figure 11.

After 85 days of immersion in seawater, a thick layer of corrosion products was deposited on the surface of the sample, which, after drying, formed a fragile crust, easily removable (the A zone). During handling, a portion of the crust was partially detached (the B zone). The microphotographs from the A zone, magnified by 1000× and 5000×, respectively, indicate that at the microscopic level the crust is formed in an agglomeration of crystalline micro formations, a structure that gives the crust a great porosity.

**Figure 12:**
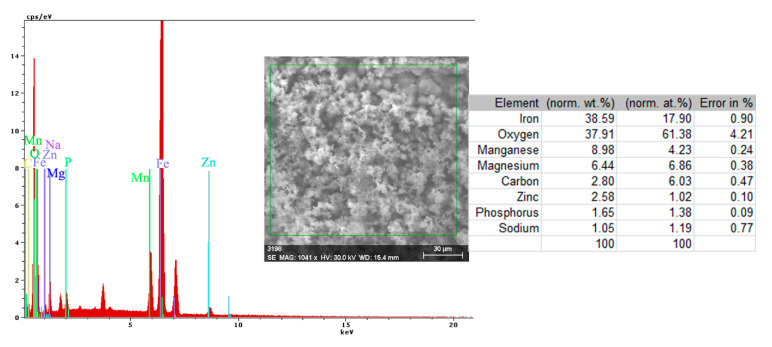
Energy spectrum and surface composition of the crust in the A zone of the PS sample maintained for 85 days in BSW.

**Figure 13:**
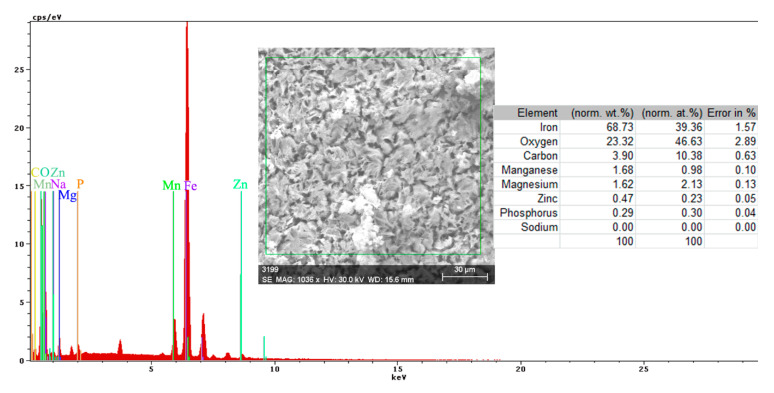
Energy spectrum and surface composition of the crust in the B zone of the PS sample maintained for 85 days in BSW.

The energy spectrum and surface composition are shown in Figure 12 for the A zone and in Figure 13 for the B zone.

**Figure 14:**
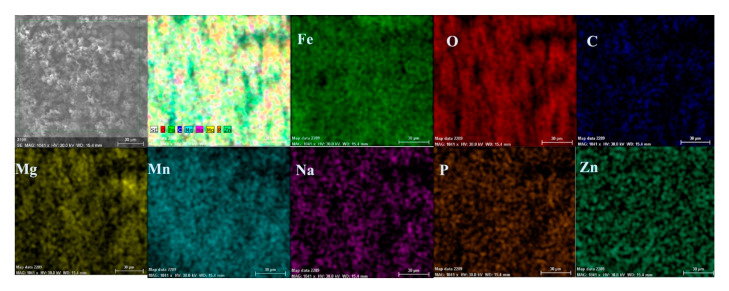
Elemental distribution in the crust on the PS surface, after 85 days of immersion in seawater.

In the A zone, the crust had a complex composition, in which the amount of iron was relatively small and the amount of oxygen was much higher than that corresponding to iron oxides or hydroxy-oxides. This means that the crust was not only made up of iron oxides. The composition of the crust contained carbon, magnesium, and sodium, which could come from the seawater left in the surface layer and evaporate on drying. Traces of phosphorus and zinc in the composition of the crust came from the phosphate layer, partially damaged in seawater, especially in the initial periods of immersion. The distribution of the elements in this area is shown in Figure 14.

In the B zone, from which a part of the crust came off, the amount of iron is much higher and the molar ratio oxygen: iron is 1:18 and corresponds to ferrous oxide (FeO). The other elements probably have the same origin as those in A zone.

The surface is mainly covered by oxidation products, Figure 14, with areas covered by thicker oxides, Figure 14 (O). Other compounds based on Mg, Mn, P, and Zn are observed on the surface. The oxides distribution is homogeneous on the entire exposed surface. The distribution of C, O, and Na suggests the formation of complex compounds such as carbonate (carbonate ${\mathrm{CO}}_{3}^{2-}$ with sodium ion to form sodium bicarbonate (NaHCO_3_) and other bicarbonates than in sodium carbonate (Na_2_CO_3_) and other carbonates). The corrosion resistance of the sample depends on the quality of the phosphate layer and the covering percentage (any area of the substrate exposed to direct corrosion environment will facilitate the corrosion).

### 3.3. Effects of Immersing the Phosphate and Painted C45 (PPS) Sample in Seawater

**Figure 15:**
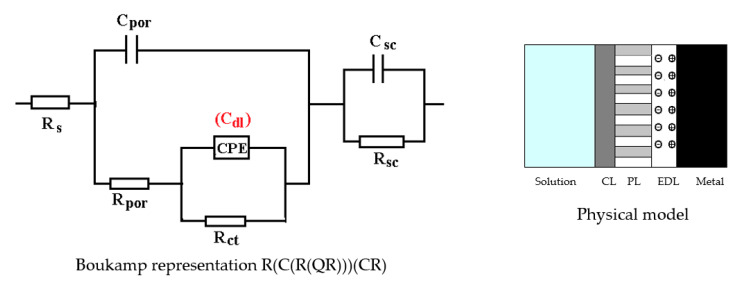
The equivalent circuit for PPS sample, after 1 h of immersion in BSW.

The C45 phosphate samples were painted and dried prior immersion in Black Sea water for 36 days and 78 days and analyzed by electrochemical impedance spectroscopy and scanning electron microscopy with energy dispersive spectroscopy. The surface microstructure at the initial contact with seawater was evaluated after 1 h of immersion. The data adjustment was made using the equivalent circuit in Figure 15.

The structure of the surface layer identifies double-electric layer (EDL)—formed by polarizing the metal surface and characterized by R_ct_ and CPE, a porous layer (PL)—representing the porous phosphating layer—characterized by R_por_ and C_por_, respectively, a compact layer (CL)—representing the paint film—characterized by the R_SC_ and C_SC_ circuit elements. The values of the circuit elements, evaluated with the ZSimp Win software, are presented in Table 6.

The analysis of the results in this table leads to the following observations:

- The chosen equivalent circuit describes very well the experimental data as indicated by the relative error values: χ^2^ = 7.2 × 10^−5^, impedance measurement error percentage ε_z_ = 0.846% and the relative percentage errors for each circuit element ε_ec_ < 5%;
- Although the geometry of the measuring cell (distance between electrodes, sample surface) was the same as in the previous measurements, the resistance of the electrolyte (Rs) is approximately three times higher (38.67 for C45 and 0.41 for SP). This is because of the actual resistance of the liquid column in addition to the resistance of the phosphate layer is also added the resistance of the paint film, which is very slightly electrically conductive;
- The resistance of the porous layer (phosphating layer) is low and is practically equal to that found for the PS/BSW system, where only the phosphate layer is present (R_ext_ ≡ R_por_ = 11.04 Ω·cm^2^);
- Due to the shields produced by the phosphate layer and the paint layer, the charge transfer resistance (R_ct_) is very high, so the corrosion resistance is good.

**Figure 16:**
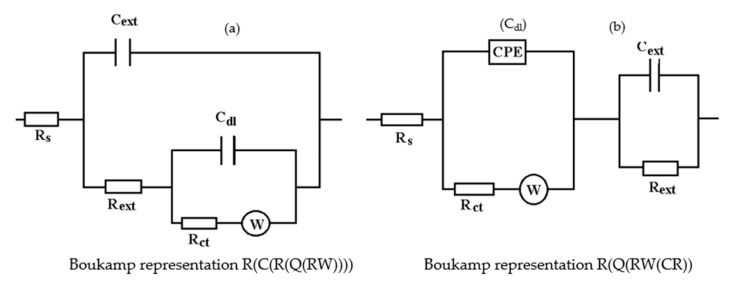
The equivalent circuits used to model EIS data in samples immersed in seawater for (**a**) 36 days or (**b**) 78 days.

**Figure 17:**
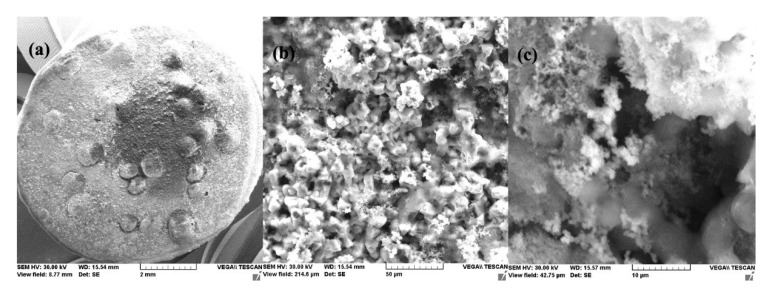
SEM microphotographs for the PPS sample maintained for 78 days in seawater (**a**) 100×, (**b**) 1000×, and (**c**) 5000×.

The equivalent circuits used for processing the electrochemical impedance spectroscopy data obtained for the painted samples immersed for a long time in seawater are presented in Figure 16 and Figure 17, and the values of the parameters of the circuit elements in Table 7. (Note: the two circuits are equivalent and express the same physical state).

The assumption of the formation of the unit layer seems to be confirmed by the fact that the resistance of this layer is much higher than the sum of the resistances of the phosphate layer and the paint layer. (R_por_ + R_SC_ = 10.8 + 21.7 = 32.5).

The resistance of the electrolyte increases to the initial moments and the immersion time in solution is proportional, as a consequence of the clogging of the pores and the deposits of the reaction products.

After the periods of immersion in the solution, it was necessary to introduce the Warburg impedance, which takes into account the diffusion of charges through the product layer. The surface state of the PPS sample maintained for 78 days in seawater is presented in Figure 16, and shown through SEM microphotographs in Figure 17, showing a corroded surface with a thick layer of compounds.

**Figure 18:**
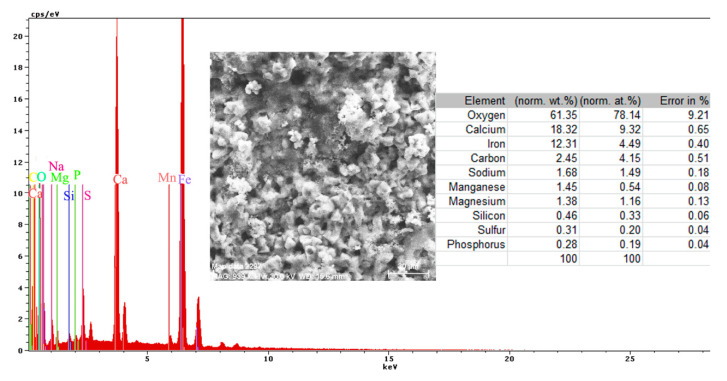
Energy spectrum and surface composition for the crust formed on the PPS surface, after 78 days of immersion in BSW.

Mainly, the compounds are based on oxides, salts, and carbonates (Figure 18). There are two different corroded areas on the surface, one of them influenced by the gas release under the oxides layer. The main elements identified on the surface (Figure 18) are O, Ca, Fe, C, Na, Mn, Mg, Si, S, and P, and emerged from the substrate and immersion solution. The stability of the material surface and the beginning of the degradation process depend on the complex layer of compounds.

The complexity of the chemical composition result is given by the multiple structures of the surface (metallic substrate, phosphate layer, and paint cover) in contact with the immersion liquid. The presence of oxygen on the surface is highly based on its presence in the paint and from the oxides formed on the surface. The presence of calcium is due to the paint scraps that are mixed with the oxides of the substrate, but also can be explained by the compounds passing from the immersion solution (BSW that contains 146 mg/dm^3^ of Ca^2+^ component).

Regarding the sulfur element, the paint materials are S free, as identified on the surface based on the compounds formed between the iron-based oxides, as well from the sea solution that has 1305 mg/dm^3^ of SO_4_^2−^. The magnesium element, as a Mg^2+^ (548 mg/dm^3^) component, is part of the BSW and passes to the surface as oxide compound or complex compound with zinc (Figure 18).

**Figure 19:**
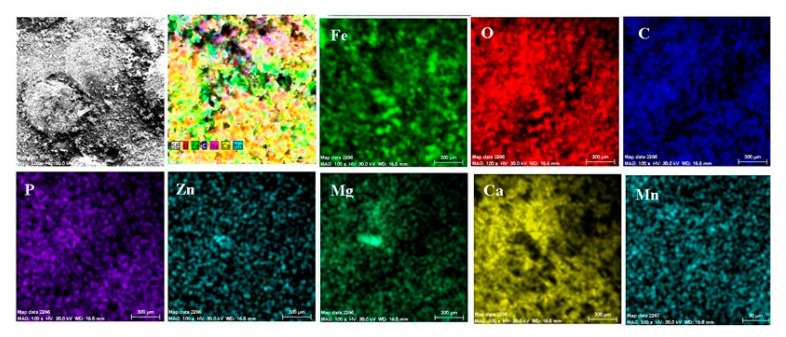
Elemental distribution in the crust on the PPS surface, after 78 days of immersion in seawater.

A part of the identified chemical elements distribution is presented in Figure 19. The presence of iron is strong in some areas of distribution (Figure 18), which means that both the paint and phosphate layer were penetrated in that part by the corrosion, and the substrate is in straight contact with the liquid environment. The exposed iron areas are near the gas release bubbles.

In the near future, these bubbles will become corrosion pits. During applications of steel metal elements in seawater, two principal arrangements of the corrosion speed-up process appeared: firstly, the old passivation surface layer, represented by partial products, was removed due to the reduction reactions of insoluble ferrous compounds to soluble ferrous ions; secondly, the anodic dissolution rate of steel was promoted because of bio-oxidation of cathodic hydrogen induced by Fe-reducing bacteria existent in seawater.

The calcium ions are specific to the paint layer and the zinc and phosphorus ions are specific to the phosphate layer. As expected, the area from which part of the crust came off is mainly the layer of paint on which a small amount of corrosion product is deposited (Fe(OOH)).

From elemental distribution, we observed an entire surface corroded (oxygen general spread) with areas of the penetrated paint layer. BSW is an extremely corrosive environment and affects most of the surface areas. From the calcium signal, Figure 19, we can observe an unaffected part of the layer where the integrity of the paint is not compromised. The differences of the corrosion resistance are given by the quality of the material’s surface and the deposition procedure parameters of the phosphate layer or paint.

Table 8 shows the corrosion rate of the samples calculated according to the method presented in Jafari H. et al., 2011 [35]. Taking into account that the basic material of the samples is carbon steel, the corrosion rate was calculated using its density (7.8 g/cm^3^), molar mass (56 g/mol) and valence (2).

As can be seen from the table, the trend of the calculated values is in accordance with the results obtained by EIS and with the stated hypotheses regarding the analyzed materials.

## 4. Conclusions

The immersion behavior, in salt water, of carbon steel, phosphate carbon steel, and phosphate and painted carbon steel was studied. Based on the experimental results, the following conclusions can be highlighted:

C45 sample:

- The immersion time increase results in a significant decrease of the R_ct_ value, from 588 cm^2^ to 155 Ω∙cm^2^, respectively, 130 Ω∙cm^2^; this aspect indicates a decrease of the corrosion rate with the immersion periods.
- After 1 h of immersion, on the surface of the C45 sample were absorbed ions from saltwater or insoluble corrosion products creating a discontinuous layer. Over time, the thickness of the products layer increase acting as a screen causing the charge transfer resistance decrease.
- The EDS spectra and quantitation data indicate the presence of the iron and oxygen on the C45 surface for all the immersion times. For the C45 sample, immersed for only 1 h, the EDS spectra show small amounts of manganese and silicon, which confirm the hypothesis above.
- For the other sample, the EDS data indicate only the presence of iron and oxygen along with small amounts of carbon. This layer has a protective role for a while and after that, the degradation of the material occurs based on a continuous process of corrosion.
- The FT-IR spectrum confirms the presence of the iron oxyhydroxide on the C45 surface.

Phosphate C45 sample:

- After immersion, the surface of the samples indicated a bi-layer structure (the phosphate layer and the corrosion products layer).
- After 43 days immersion, the phosphate layer is partially damaged, widening its pores, but after 85 days, the resistance of the outer layer increases due to the thickness increase of the product layer.
- The composition of the crust form on the surface sample present not just iron and oxygen, but also phosphorus and zinc from the phosphate layer and carbon, magnesium, and sodium from the seawater composition.

Phosphate and pained C45 sample:

- The EIS data reveal that the phosphate layer resistance is equal compared with the value obtained for the PS sample.
- Even the saltwater damaged the paint layer; this sample has good corrosion resistance.

## Figures and Tables

**Table 1:** Chemical composition of the experimental low carbon steel.

Element	C	Mn	P	Cu	Cr	Si	Fe
**Percent (wt.%)**	0.45	0.98	0.02	0.15	0.17	0.22	balance

**Table 2:** Chemical composition of phosphate solution.

Name of the Active Substance	Quantity
Sodium hydroxide (NaOH)	0.75 g
Sodium azotite (NaNO_2_)	0.45 g
Sodium tripolyphosphate (Na_5_P_3_O_10_)	0.05 g
Phosphoric acid (H_2_PO_4_)	10.00 mL
Nitric acid (HNO_3_)	4.00 mL
Zinc (Zn)	3.50 g
iron (Fe)	0.038 g

**Table 3:** Variation of the corrosion environment pH with the immersion time of the samples.

Corrosion Environment	Immersion Time (Days)	C45	PS	PPS
**pH of BSW**	Initial	6.15	6.15	6.15
	45 or 36	6.25	7.30	7.22
	85 or 78	6.71	7.57	7.76

45 days—C45 and PS; 36 days—PPS; 85 days—C45 and PS; 78 days—PPS.

**Table 4:** Equivalent circuit element values for C45 immersed for various periods in BSW.

Immersion Time	R_s_ Ω·cm^2^	CPE		R_ct_ Ω·cm^2^	R_ext_ Ω·cm^2^	W Ss^1/2^/cm^2^	10^3^ × χ^2^	ε_z_
		Q Ss^n^/cm^2^	n					
43 days	39.470	6.92 × 10^−3^	0.749	155	5.01	-	2.439	4.94
85 days	43.590	1.25 × 10^−2^	0.772	130	145.90	0.065	0.090	0.95

**Table 5:** Equivalent circuit element values for PS immersed for various periods in BSW.

Immersion Time	R_s_ Ω·cm^2^	CPE		R_ct_ Ω·cm^2^	R_ext_ Ω·cm^2^	C_ext_ µF/cm^2^	W Ss^1/2^/cm^2^	10^3^ × χ^2^	ε_z_
		Q Ss^n^/cm^2^	n						
1 h	40.41	2.51 × 10^−4^	0.613	883.00	11.04	2.870	-	0.694	2.44
43 days	39.47	5.10 × 10^−3^	0.866	334.20	12.35	2.667	-	0.155	1.24
85 days	41.86	6.63 × 10^−3^	0.732	280.60	14.55	147	0.042	0.036	0.55

**Table 6:** Equivalent circuit parameters R (C (R (QR))) (CR) for PPS in seawater in the initial moments.

-	R_s_ Ω·cm^2^	C_por_ µF/cm^2^	CPE		R_ct_ Ω·cm^2^	R_SC_ Ω·cm^2^	C_SC_ µF/cm^2^	10^3^·× χ^2^	ε_z_
			Q Ss^n^/cm^2^	n					
-	115	0.307	6.81 × 10^−4^	0.68	1121	21.70	1200	0.072	0.846
ε_EC_	1.36	3.010	0.66	0.38	334.20	4.61	5.15	-	-

**Table 7:** Equivalent circuit element values for PPS immersed 36 and 78 days in BSW.

Immersion Time	R_s_ Ω·cm^2^	CPE		R_ct_ Ω·cm^2^	R_ext_ Ω·cm^2^	C_ext_ µF/cm^2^	W Ss^1/2^/cm^2^	10^3^·× χ^2^	ε_z_
		Q Ss^n^/cm^2^	n						
36 days	315.40	C_dl_ = 1.73 µF/cm^2^		176.50	266.90	0.022	5.96 10^-3^	4.13	6.43
78 days	385.70	6.63 × 10^−3^	0.732	145.80	184.60	0.267	6.42 10^-3^	1.75	2.64

**Table 8:** The corrosion rate of the studied samples.

Corrosion Rate	Immersion Time	C45	PS	PPS
**CR (** **mpy)**	1 h	0.027	0.011	0.008
	45 (36) days	0.062	0.029	0.022
	85 (78) days	0.036	0.034	0.030

## Data Availability

The data presented in this study are available on request from the corresponding author.

## References

[1] Odusote J.K., Ajiboye T.K., Rabiu A.B. (2012). Evaluation of Mechanical Properties of Medium Carbon Steel Quenched in Water and Oil. J. Miner. Mater. Charact. Eng..

[2] Boumerzoug Z., Derfouf C., Baudin T. (2010). Effect of Welding on Microstructure and Mechanical Properties of an Industrial Low Carbon Steel. Engineering.

[3] Panda A., Duplak J., Hatala M., Krenicky T., Vrabel P. (2016). Research on the Durability of Selected Cutting Materials in the Process of Turning Carbon Steel. MM Sci. J..

[4] Karavaeva M.V., Nurieva S.K., Zaripov N.G., Ganeev A.V., Valiev R.Z. (2012). Microstructure and mechanical properties of medium-carbon steel subjected to severe plastic deformation. Met. Sci. Heat Treat..

[5] Kimapong K., Poonayom P., Wattanajitsiri V. (2016). Microstructure and wear resistance of hardfacing weld metal on JIS-S50C carbon steel in agricultural machine parts. Adv. Eng. Forum..

[6] Burduhos-Nergis D.P., Baciu C., Vizureanu P., Lohan N.M., Bejinariu C. (2019). Materials types and selection for carabiners manufacturing: A review. Proceedings of the IOP Conference Series: Materials Science and Engineering.

[7] Bejinariu C., Darabont D.C., Baciu E.R., Georgescu I.S., Bernevig-Sava M.A., Baciu C. (2017). Considerations on applying the method for assessing the level of safety at work. Sustainability.

[8] Hamidinejad S.M., Kolahan F., Kokabi A.H. (2012). The modeling and process analysis of resistance spot welding on galvanized steel sheets used in car body manufacturing. Mater. Des..

[9] Sekban D.M., Aktarer S.M., Xue P., Ma Z.Y., Purcek G. (2016). Impact toughness of friction stir processed low carbon steel used in shipbuilding. Mater. Sci. Eng. A.

[10] Usher K.M., Kaksonen A.H., Cole I., Marney D. (2014). Critical review: Microbially influenced corrosion of buried carbon steel pipes. Int. Biodeterior. Biodegrad..

[11] Nedeff V., Bejenariu C., Lazar G., Agop M. (2013). Generalized lift force for complex fluid. Powder Technol..

[12] Nica P.E., Agop M., Gurlui S., Bejinariu C., Focsa C. (2012). Characterization of aluminum laser produced plasma by target current measurements. Jpn. J. Appl. Phys..

[13] Bacaita E.S., Bejinariu C., Zoltan B., Peptu C., Andrei G., Popa M., Magop D., Agop M. (2012). Nonlinearities in drug release process from polymeric microparticles: Long-time-scale behaviour. J. Appl. Math..

[14] Dwivedi D., Lepková K., Becker T. (2017). Carbon steel corrosion: A review of key surface properties and characterization methods. RSC Adv..

[15] Manna M. (2009). Characterisation of phosphate coatings obtained using nitric acid free phosphate solution on three steel substrates: An option to simulate TMT rebars surfaces. Surf. Coat. Technol..

[16] Abdalla K., Rahmat A., Azizan A. (2014). Influence of activation treatment with nickel acetate on the zinc phosphate coating formation and corrosion resistance. Mater. Corros..

[17] Burduhos-Nergis D.-P., Vizureanu P., Sandu A.V., Bejinariu C. (2020). Phosphate Surface Treatment for Improving the Corrosion Resistance of the C45 Carbon Steel Used in Carabiners Manufacturing. Materials.

[18] Etteyeb N., Sanchez M., Dhouibi L., Alonso C., Andrade C., Triki E. (2006). Corrosion protection of steel reinforcement by a pretreatment in phosphate solutions: Assessment of passivity by electrochemical techniques. Corros. Eng. Sci. Technol..

[19] Darband G.B., Aliofkhazraei M. (2017). Electrochemical phosphate conversion coatings: A review. Surf. Rev. Lett..

[20] Burduhos-Nergis D.-P., Bejinariu C., Toma S.-L., Tugui A.-C., Baciu E.-R. (2020). Carbon steel carabiners improvements for use in potentially explosive atmospheres. MATEC Web Conf..

[21] Bejinariu C., Burduhos-Nergiș D.P., Cimpoeșu N., Bernevig-Sava M.A., Toma Ș.L., Darabont D.C., Baciu C. (2019). Study on the anticorrosive phosphated steel carabiners used at personal protective equipment. Qual. Access Success.

[22] Schmidt D.P., Shaw B.A., Sikora E., Shaw W.W. (2006). Corrosion protection assessment of barrier properties of several zinc-containing coating systems on steel in artificial seawater. Corrosion.

[23] Guenbour A., Benbachir A., Kacemi A. (1999). Evaluation of the corrosion performance of zinc-phosphate-painted carbon steel. Surf. Coat. Technol..

[24] Asadi V., Danaee I., Eskandari H. (2015). The effect of immersion time and immersion temperature on the corrosion behavior of Zinc phosphate conversion coatings on carbon steel. Mater. Res..

[25] Möller H., Boshoff E., Froneman H. (2006). The corrosion behaviour of a low carbon steel in natural and synthetic seawaters. J. S. Afr. Inst. Min. Metall..

[26] Burduhos-Nergis D.P., Vizureanu P., Sandu A.V., Bejinariu C. (2020). Evaluation of the corrosion resistance of phosphate coatings deposited on the surface of the carbon steel used for carabiners manufacturing. Appl. Sci..

[27] Díaz B., Freire L., Mojío M., Nóvoa X.R. (2015). Optimization of conversion coatings based on zinc phosphate on high strength steels, with enhanced barrier properties. J. Electroanal. Chem..

[28] Burduhos-Nergis D.-P., Sandu A.-V., Burduhos-Nergis D.-D., Darabont D.-C., Comaneci R.-I., Bejinariu C. (2019). Shock Resistance Improvement of Carbon Steel Carabiners Used at PPE. MATEC Web Conf..

[29] Mobin M., Shabnam H. (2011). Corrosion behavior of mild steel and SS 304L In presence of dissolved nickel under aerated and deaerated conditions. Mater. Res..

[30] Nagiub A.M. (2005). Evaluation of Corrosion Behavior of Copper in Chloride Media Using Electrochemical Impedance Spectroscopy (EIS). Port. Electrochim. Acta.

[31] Chen M., Du C.Y., Yin G.P., Shi P.F., Zhao T.S. (2009). Numerical analysis of the electrochemical impedance spectra of the cathode of direct methanol fuel cells. Int. J. Hydrog. Energy.

[32] Cao C., Zhang J. (2002). Introduction of Electrochemical Impedance Spectroscopy.

[33] Xu X., Liu S., Smith K., Cui Y., Wang Z. (2020). An overview on corrosion of iron and steel components in reclaimed water supply systems and the mechanisms involved. J. Clean. Prod..

[34] Boily J.F., Felmy A.R. (2008). On the protonation of oxo- and hydroxo-groups of the goethite (α-FeOOH) surface: A FTIR spectroscopic investigation of surface O-H stretching vibrations. Geochim. Cosmochim. Acta.

[35] Jafari H., Idris M.H., Ourdjini A., Rahimi H., Ghobadian B. (2011). EIS study of corrosion behavior of metallic materials in ethanol blended gasoline containing water as a contaminant. Fuel.

